# Help Wanted

**Published:** 1897-02

**Authors:** 

**Affiliations:** Texas


### Correspondence.

### Help Wanted.

——— TEXAS, JANUARY 2, 1897.

*Editors Texas Medical Journal:*

I write you to-day, and ask if you will publish in your valuable journal a sure cure for youthful errors, etc. Have been a masturbator for four or five years, and cannot break myself of this ruinous habit. Have never had any "quacks" to give me anything, for I want only the best information. This will be a great help to me, and perhaps others, too, who are not willing for anyone to know their condition. Am 21 years old, and have organs about like a 12-year-old boy. If this is not asking too much, please give a little of your space to this subject in your next issue. Give a prescription, and I will try it. Am confident that Dr. P. and several others will write something on this subject. I don't want you to be at any expense for this, but if you possibly can, please give me help.

Trusting this will not be too much to ask of you, and thanking you in advance for your trouble, I wait, hoping this will be carefully noted.

AN UNFORTUNATE YOUNG MAN.

[See Editorial Department. There is one "sure cure" for such cases. If any of the JOURNAL readers have had successful experience in *treating* this condition, we will be pleased to hear from them, and will publish their replies for the benefit of this young man.—ED.]

